# The Role of Glucagon-like Peptide 1 Receptor Modulators for the Management of Dumping Syndrome in Patients Who have Undergone Metabolic and Bariatric Surgery: a Scoping Review

**DOI:** 10.1007/s11695-026-08587-4

**Published:** 2026-03-19

**Authors:** Madeline Rogers-Seeley, Natalie Guiney, Khang Duy Ricky Le

**Affiliations:** 1 Royal Melbourne Hospital, Melbourne, Australia; 2 Deakin University, Burwood, Australia

**Keywords:** Glucagon-like peptide 1 agonists, GLP-1, GLP-1 RA, Dumping syndrome, Metabolic and bariatric surgery

## Abstract

**Background:**

Dumping syndrome is a common complication following metabolic and bariatric surgery (MBS) and is associated with significant morbidity and impaired quality of life. While glucagon-like peptide-1 receptor modulators (GLP-1 RMs) are established therapies for obesity, their role in the management of dumping syndrome remains unclear. Existing literature is limited and has largely focused on postprandial hypoglycaemia as a surrogate for late dumping syndrome.

**Objective:**

To map the current evidence regarding the safety and efficacy of GLP-1 RMs in the management of dumping syndrome following MBS, recognising dumping syndrome as a broader clinical entity encompassing symptoms, quality of life, and glucose stabilisation.

**Methods:**

A scoping review was conducted in accordance with Joanna Briggs Institute methodology and reported using PRISMA-ScR guidelines. A comprehensive search of multiple databases was performed to identify peer-reviewed clinical studies evaluating GLP-1 RMs for dumping syndrome after MBS. Data were extracted on study design, patient characteristics, type of dumping syndrome, GLP-1 RM regimen, and clinical outcomes.

**Results:**

Fifteen studies involving 107 patients were included - predominantly case reports and case series. GLP-1 receptor agonist therapy was associated in these cases with reduction or resolution of hypoglycaemic episodes and improvement in dumping-related symptoms. Limited evidence also suggested improvements in quality of life. The GLP-1 receptor antagonists in small studies showed some stabilisation of postprandial glucose profiles and attenuating exaggerated insulin responses. Adverse effects were poorly recorded but appeared well tolerated in short term studies.

**Conclusions:**

Current evidence suggests a potential role for GLP-1 RMs in the management of both early and late dumping syndrome following MBS. However, the evidence base is limited by small sample sizes, heterogeneous diagnostic criteria, and variable outcome reporting. High-quality, prospective studies using standardised definitions and diagnostic approaches are required to clarify efficacy, safety, and long-term outcomes.

## Introduction

Obesity is defined as a Body Mass Index (BMI) >30 kg/m^2^ by the World Health Organisation (WHO) and is a complex interaction of factors requiring multi-faceted and sustained management [1–3]. It is a well-known risk factor for many serious chronic conditions [2, 4]. Bariatric surgery or metabolic and bariatric surgery (MBS), induces sustained weight loss and reduces obesity-related complications over the long term. In Australia, data from 2023 describes 15 985 primary MBScompleted in the same year and the incidence of these procedures continues to rise in Australia [5, 6]. While Glucagon-Like Peptide 1 modulators (or GLP1-RMs) such as those with agonistic mechanisms of action have emerged as effective agents for weight management, MBS remains the most effective intervention for Class II (BMI 35–39.9.9) and Class III (BMI 40+) obesity [6]. However, dumping syndrome is a common complication after surgery that may theoretically be influenced by GLP-1 physiology.

Dumping syndrome is estimated to occur in up to 40% of patients undergoing Roux-En-Y Gastric bypass (RYGB), and MBS is the most common cause [7, 8]. Furthermore, these values differ depending on the other MBS options being undertaken; for instance, the estimated incidence for patients who undergo one-anastomosis gastric bypass is approximately 42.9% and around 15.6% for those who undergo sleeve gastrectomy [9]. These values however differ depending on jurisdiction, with others areas reporting incidences that are significantly less [10]. Dumping syndrome itself is is divided into early and late [9, 11]. Reduction in volume of the stomach allows food, particularly carbohydrate rich food, to transit into the small intestines with less regulation, and pending anatomical resection removes the pylorus function of the stomach. Early dumping syndrome is postulated to be due to hyperosmolar food contents causing a fluid shift from gastrointestinal vasculature into the lumen [9]. Gastrointestinal symptoms of this include abdominal pain and distention, nausea, and diarrhoea, and vasomotor effects can include diaphoresis, palpitations, weakness and flushing [9, 11]. Late dumping syndrome is thought to be from a rapid spike in blood glucose levels from this causing an excessive amount of insulin being secreted from the pancreas and an excessive GLP-1 response resulting in a hypoglycaemic episode 1–3 h after the meal [9]. Late dumping syndrome can cause significant morbidity and mortality, with sequelae of hypoglycaemia including fatigue, confusion, diaphoresis but also as severe as seizures, injury from syncopal events, and even death [9, 11]. Quality of life is significantly impacted by both early and late, and management is difficult, usually given in stepwise approach of dietary, medical or surgical re-intervention options [12]. Consensus was gained on defining early dumping syndrome symptomatically and late by reactive hypoglycaemia 1–3 h after a meal (both in the presence of the relevant surgical history) [9, 11]. With purpose-build scoring systems being debunked as useful, and mixed diagnostic criteria being used in case studies, this makes potential novel interventions difficult to study. Furthermore, there were no agreed diagnostic tests for dumping syndrome – as described in the limitations of the studies included in this scoping review of GLP-1 RMs [9, 11, 13].

Current literature regarding the use of GLP-1 RMs for dumping syndrome is limited, with one systematic review including 31 patients across heterogeneous study designs and assessing symptomatic postprandial hypoglycaemia as a surrogate investigation for late dumping syndrome after MBS [13]. Therefore, the efficacy and safety of GLP-1 RMs for use in dumping syndrome, despite the enabling theoretical physiological basis for these medications, remains poorly characterised. This scoping review therefore aims to provide an up-to-date analysis and synthesis of the current and emerging evidence evaluating the use of GLP-1 RMs in dumping syndrome experiences by the patient who underwent MBS. In doing so, this review seeks to highlight future avenues for research and potential translation of these medications for treatment guidelines related to dumping syndrome.

## Methods

### Scoping Review Approach

This scoping review was conducted in accordance to the guidelines set by the Joanna Briggs Institute (JBI) [14]. Reporting of this review was performed as per the Preferred Reporting Items for Systematic Reviews and Meta-Analyses extension for Scoping Reviews (PRISMA-ScR) guidelines [15].

## Literature Search Strategy

A comprehensive literature search was conducted on Medline, Embase, Emcare, The University of Melbourne full-text journals repository, Google Scholar and Evidence-Based Medicine (EBM) reviews (including the following databases; Cochrane Database of Systematic Reviews, American College of Physicians (ACP) Journal Club, Database of Abstracts of Reviews of Effects, Cochrane Clinical Answers, Cochrane Central Register of Controlled Trials, Cochrane Methodology Register, Health Technology Assessment and the National Health Service (NHS) Economic Evaluation Database) on May 05, 2025. Where appropriate, additional articles were captured from the reference lists of relevant articles. The population is humans, the concept is clinical studies evaluating the use of GLP-1 RMs for dumping syndrome in patients who have undergone MBS and the context was peer-reviewed papers from any scientific field. The complete search strategy is available in the appendix.

### Eligibility Criteria

Originally, peer-reviewed articles available in the English language that evaluated the use of GLP-1 RMs for dumping syndrome in patients who have undergone MBS were considered for inclusion in this scoping review. Furthermore, due to the fundamental premise of this scoping review to exploring the foundational evidence for GLP-1RMs in dumping syndrome, study designs including conference papers, abstracts and presentations, editorials and letters to the editor. Articles were excluded if they non-human trials or were secondary research methods including meta-analyses, systematic or non-systematic reviews, scoping reviews, narrative review and literature reviews. Articles were also excluded if they looked at post-prandial hypoglycaemia in patients who have not undergone any bariatric surgical procedure or did not evaluate any of the outcomes of interest.

### Outcomes

Outcomes of interest were related to the safety and efficacy of GLP-1 RMs for use in patients who experience dumping syndrome following MBS. Furthermore, due to the iterative nature of scoping review methodology and the fundamental aim of this paper in characterising the emerging evidence for GLP-1RMs in dumping syndrome, additional relevant outcomes related to use of these medications for dumping syndrome were also obtained and descriptively reported where relevant.

### Literature Screening

Articles captured from the literature search were uploaded onto the Covidence platform (Cochrane, United Kingdom). Following the automatic de-duplication of articles, screening by title and abstract was performed by two independent investigators (MRS, KL). Articles with insufficient information or data progressed to full-text analysis. Full-text analysis was performed by the same two independent investigators. Disagreement during these processes was resolved by consensus.

### Data Extraction

Included articles were first extracted for bibliometric and methodological parameters including author, year and country of publication, study design, demographic factors (patient age, sex, sample size, body mass index), specific GLP-1 RM used (medication, dose, regimen), type of dumping syndrome being managed (early, late, not specified), duration of follow-up and clinical outcomes including symptom improvement, glycaemic control and adverse effects. Additional relevant findings from each study were also captured.

### Data Synthesis

Data were extracted and synthesised in summary tales and figures according to relevant themes including efficacy outcomes and safety outcomes. Given the scoping review methodology, no meta-analysis or statistical analysis was conducted. Furthermore, no formal assessment of study quality was performed, in keeping with the objective to map the current landscape of evidence.

## Results

### Literature Search Results

**Fig. 1:**
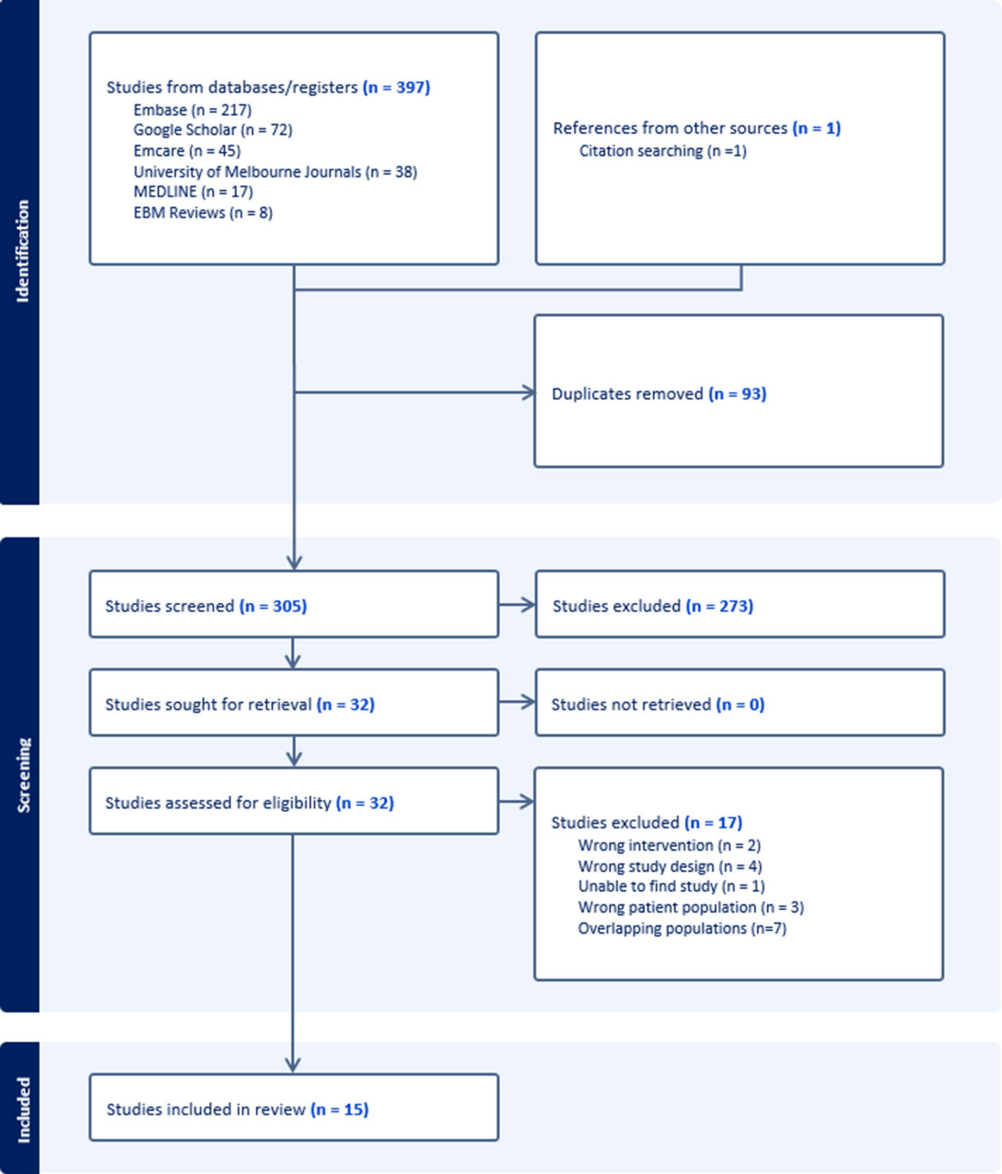
PRISMA flowchart depicting search strategy

Our literature search identified 397 unique articles, with an additional article found via manual citation searching. Following screening by title and abstract, 32 articles progressed to full-text analysis. Of these, 15 articles satisfied the eligibility criteria and were included in this scoping review (Fig. 1).

### Overview of Studies

15 studies evaluated the role of GLP-1 RMs for use in patients with dumping syndrome post-MBSand were included in this scoping review [16–30] (Table 1).

**Table 1:** Overview of Studies

Author	Year of publication	Country of publication	Type of study	Type of surgery	Class of GLP1-RM	Number of participants	Sex (M, F)	Age
Alsayed Hasan	2023	USA	Case series	Laparoscopic roux-en-y	GLP-1 receptor agonist	3	0, 3	39.7 (mean)
Arevalo	2024	USA	Case study	Gastric sleeve	GLP-1 receptor agonist	1	0, 1	47
Brown	2018	USA	Case study	Roux-en-y gastric bypass	GLP-1 receptor agonist	1	0, 1	45
Craig	2017	USA	Double-blinded crossover study	Roux-en-y gastric bypass	GLP-1 receptor antagonist	16	0, 16	46.6
Craig	2021	USA	Randomised, placebo-controlled crossover trial	NR	GLP-1 receptor antagonist	18	0, 18	44.3
Dorado	2017	Colombia	Case report	Laparoscopic roux-en-y gastric bypass	GLP-1 receptor agonist	1	0, 1	41
Fiore	2024	Italy	Case report	Gastric bypass	GLP-1 receptor agonist	1	0, 1	31
Leuz	2021	Germany	Case report	Initial Billroth II operation for perforated gastric ulcer, later converted to Billroth I (partial gastrectomy	GLP-1 receptor agonist	1	1,0	60
Lorde	2023	United Kingdom	Case report	Gastric bypass	GLP-1 receptor agonist	1	0,1	36
May	2023	United Kingdom	Case report	Roux-en-Y gastric bypass	GLP-1 receptor agonist	1	0,1	50
Abrahamsson	2013	Sweden	Case series	Gastric bypass	GLP-1 receptor agonist	5	0, 5	44.4 (mean)
Salehi	2014	USA	Case control	Gastric bypass	GLP-1 receptor antagonist	9 symptomatic, 7 asymptomatic, 8 controls (no operations)	0/9, 4/3, 1/7	44.6, 47.6, 36.1 (mean)
Shaghouli	2021	Kuwait	Case series	NR	GLP-1 receptor agonist	27	2, 25	44.64 +/- 10.2
Stier	2015	Germany	Conference poster of case series	NR	GLP-1 receptor agonist	6	NR	46 +/- 11.26
Stortz	2024	USA	Case report	Gastric band	Dual GLP-1 receptor agonist and glucose-dependent insulinotropic polypeptide (GIP)	1	0, 1	46

Of these, 12 examined cases using GLP-1 receptor agonists, two used GLP-1 receptor antagonists, and one used a dual GLP-1 receptor agonist and glucose-dependent insulinotropic polypeptide (GIP). The majority were published in the United States of America (7/15, 46.67%) followed by Germany (2/15, 13.33%), the United Kingdom (2/15, 13.33%), Sweden, Kuwait, Italy and Colombia (all 1/15, 6.67%). The most common study design was case reports and case studies (8/15, 53.33%) followed by case series (4/15, 26.67%). Overall, these studies encompassed 107 individual patients. Despite variable reporting, most studies report a predominance of female participants with age ranging from 31 to 60 years, with an estimated mean around 44.2 years of age. The most common bariatric surgery performed was a gastric bypass (including Roux-en-Y gastric bypass) (9/15 studies, 60.00%). There was incomplete reporting of body mass index and weight lost variables.

### Outcomes

15 studies involving 107 patients were assessed which suggested some benefit including potential resolution or reduction in hypoglycaemic episodes and/or symptoms and/or glucose stabilising effects in all cases treated with GLP-1 RMs (Table 2). They were then separated in areas of interest into the modulation effect on GLP-1 – either receptor agonism, antagonism, or combined agonist with GIP.

**Table 2:** The use of glucagon-like peptide 1 receptor agonists in dumping syndrome

Author(s)	Year of publication	BMI pre	BMI post	Weight lost	Type of dumping syndrome	Diagnosis of dumping syndrome	Class of GLP-1 RM used	GLP-1 RM used	Dosing regimen	Concurrent treatments	Outcome	Adverse effects
Alsayed Hasan	2023	45.7 (mean)	29.9 (mean)	NR (2/3 recorded)	Late	NR	GLP-1 receptor agonist	Liraglutide	Uptitration to 1.2 mg/day	Nil	Resolution of hypoglycaemia (*n* = 3)	One case didn’t alter diet and developed post prandial vomiting but when educated stopped eating with satiety and these resolved on same dosing liraglutide
Arevalo	2024	29.9	NR	NR	Late	24 h continuous glucose measurements, point of care capillary glucose measurement + symptoms	GLP-1 receptor agonist	Semaglutide	0.25 mg weekly	Acarbose and dioxazide (not tolerated, ceased prior to semagluide starting)	Resolution of hypoglycaemia no episodes in three months	NR
Brown	2018	NR	NR	3.6 kg	Late	24 h continuous glucose measurements + Symptoms (seizures with fractures)	GLP-1 receptor agonist	Exenatide	5micog daily uptitrated to 10microg daily	Acarbose, octreotide, diazoxide, small frequent low carb meals (no improvement)	One single episode in six months with no seizures	NR
Craig	2017	31	NR	NR	Late	OGTT + symptoms	GLP-1 receptor antagonist	Placebo or Ex-9 (7500pmol/kg) (also known as Avexitide)	Day 1 and Day 7	Nil	Infusion of ex-9 decreased time to peak glucose and rate of glucose decline, raising it in line to non-surgical control group. Autonomic and neuroglycopaenic symptoms were reduced also	Hypoglycaemia in the control group (not in the surgical group receiving Ex-9
Craig	2021	29.6	NR	NR	NR	Mixed meal tolerance test, (blinded) CGM, self glucose monitoring, diary of symptoms	GLP-1 receptor antagonist	Avexitide (also known as Ex-9	Placebo for 14 days followed by avexitide 30 mg twice daily and 60 mg once daily, each for 14 days in random order (either placebo then 30 mg for 2 weeks then 60 for two weeks, or placebo then 60 mg for two weeks then 30 mg for two weeks)	Nil	Compared to placebo avexitide 30 mg twice daily and 60 mg once daily raised the glucose nadir by 21% (*P* =.001) and 26% (*P* =.0002) and lowered the insulin peak by 23% (*P* =.029) and 21% (*P* =.042), corresponding to 50% and 75% fewer participants requiring rescue during MMTT, respectively. Less episodes of hypo without any increase hypergycaemic episodes.	Nil seen in 28D
Dorado	2017	40	24.3	41	Late	OGTT	GLP-1 receptor agonist	Liraglutide	NR	Lipase (for steatorrhoea)	No further hypoglycaemic episodes after 1 month, no further symptoms after 1 year	NR
Fiore	2024	NR	NR	NR	NR	Flash continuous glucose monitor	GLP-1 receptor agonist	Semaglutide	0.25 mg weekly, with increasing doses at 0.25 mg per week for 1 month and then subsequently 0.5 mg weekly	Nil	After the first few weeks, symptoms of dumping were significantly reduced with improvement of the daily glycaemic profile and resolution of hypoglycaemia (persistent up to 8 months). Time spent with blood glucose < 70 mg/dL, decreased by 12% to 4% during treatment with semaglutide 0.25 mg per week dose, up to 1% with a dose of 0.5 mg/week. - Improvement in quality of life	
Leuz	2021	NR	18.9	NR	Combined early and late	24 h continous glucose measurements	GLP-1 receptor agonist	Liraglutide	NR	Acarbose	Improved mood and quality of life. Reduced hypoglycaemic events (no levels below 40 mg/dL).	Unintentional weight loss initially (amount not reported) which stabilised with regular meals
Lorde	2023	NR	NR	NR	Late	Flash continuous glucose monitor	GLP-1 receptor agonist	Liraglutide	0.6 mg daily	Nil	Complete cessation of symptoms and hypoglycaemic events. Improved quality of life.	NR
May	2023	NR	NR	NR	Late	24 h continuous glucose measurements	GLP-1 receptor agonist	Liraglutide	0.6 mg daily uptitrated to 3 mg daily	Empagliflozin 10 mg daily	Target below range of 2.4% improved to 0% with resolution of hypoglycaemia as well as symptoms instantly. Target below range was sustained at 0% with liraglutide and empagliflozin,	NR
Abrahamsson	2013	40.25 (mean)	30.33 (mean)	47.67 kg (mean)	NR	24 h continuous glucose measurements, point of care capillary glucose measurement + symptoms	GLP-1 receptor agonist	Liraglutide	Variable range from 1.2 mg daily(*n* = 4) to 1.8 mg daily (*n* = 1)	Acarbose and nifedipine (no effect) (*n* = 1) Nifedipine, diazoxide, acarbose (no effect) (*n* = 1)	Resolution of hypoglycaemia (*n* = 5). For one patient, uptitrating dose from 1.2 mg/day to 1.8 mg/day to resolve hypoglycaemia led to nausea and headache. For those who experiences side effects, liraglutide changed to exenatide with no side effects and no return of hypoglycaemic episodes. Conclusion: GLP-1 analogues can be used in severe cases of dumping and are superior in effect to current treatments such as GI problems with glucosidase inhibitors, unwanted hormonal side effects and bile stone formation with somatostatin analogues, hypotension with calcium blockers, hypertrichosis with diazoxide.	Relapse of symptoms with dose lowering 0.6 mg/day or withdrawal of liraglutide (*n* = 2) Heart flutter, diarrhoea and therefore cessation of treatment (*n* = 1) Nausea and headache and therefore cessation of treatment (*n* = 1) Nausea and abdominal pain and therefore cessation of treatment (*n* = 1)
Salehi	2014	30.9, 33.8, 32.8 (mean)	NR	NR	NR	Meal tolerance test	GLP-1 receptor antagonist	Ex-9 (also known as Avextide)	Ex-9 one day, placebo saline next	NR	Post GB surgery who experienced hypoglycemia had more stabilized post prandial sugars than those who didn’t experience symptoms or who had no gastric surgery	
Shaghouli	2021	NR	NR	NR	Late	NR	GLP-1 receptor agonist	Liraglutide	Variable	With or without low glycemic index diet	87% of patients had reduction in hypoglycemic episodes. Of these patients, 46% had no further episodes and 54% had 1–2 episodes.	NR
Stier	2015	NR	NR	NR	Late	OGTT	GLP-1 receptor agonist	Liraglutide	0.6 mg subcutaneous liraglutide daily for 7 days then increased to 1.2 mg daily.	Nil	In absence of Liraglutide treatment, OGTT showed an early peaking of plasma glucose levels accompanied by tardy, disharmonic peaking of insulin and therefore resulting symptoms of dumping. With liraglutide (0.6 mg), peak level of insulin secretion was lowered and better in time than without liraglutide, however still a persistence of insulin level and a delayed decrease which still often led to delayed dumping symptoms due to ambiguity coordination of insulin and glucose level. With liraglutide (1.2 mg) there was better synchronized and adequate, decreased insulin level with correspondent resolution of late dumping symptoms. Conclusion: with liraglutide, symptoms resolved in 5/6 patients.	NR
Stortz	2024	45	35	25 kg	NR	24 h continuous glucose monitor	Dual GLP-1 receptor agonist and glucose-dependent insulinotropic polypeptide (GIP)	Tirzepatide	2.5 mg daily (increased to 5 mg daily for weight loss)	Nil	4 weeks after treatment, reduction in postprandial blood glucose peak from 320 mg/dL (17.8 mmol/L) to 110 mg/dL (6.1 mmol/L) noted. Time in range from 90–93% to 98%. Coefficient of variation 33.3% to 20.5%. HbA1c decreased from 5.7% to 5.4%. Symptoms resolved.	Due to insurance issues, unable to obtain tirzepatide to insurance issues for 6 weeks, symptoms returned.

### Hypoglycaemic Episodes

Eight of the 15 studies (totalling 14 patients) demonstrated no episodes of hypoglycaemia after treatment with a GLP-1 RA [16, 17, 21–26]. Three studies (35 patients) showed a reduction in hypoglycaemic events and one case series showed a mix of 1–2 episodes (47% *n* = 7), complete resolution (40% *n* = 6) and no change (13% *n* = 2), however it is important to note that of the initially recruited 25 patients in this case series only 15 responded [18, 19, 21, 28].

### Other Outcomes Measured

#### Symptoms

Other studies included the outcome of symptoms of dumping syndrome. These can include nausea and/or vomiting, abdominal distention, diarrhoea, sweating, feeling light-headed, or palpitations [31]. Seven studies (44 patients) had reduction or resolution of symptoms of dumping syndrome after the use of GLP-1 RM treatment [19–22, 24, 29, 30] Both the GLP-1 receptor agonist and 2 of the 3 antagonist modulators had subjective symptomatic relief in their outcomes (with the third antagonist case control series not reporting on symptoms as an outcome) [19, 21, 27].

#### Stabilisation of Glucose Levels

Several studies’ outcomes showed that GLP-1 RMs may have a stabilising effect on glucose levels. One crossover study of a GLP-1 receptor antagonist involving 16 people measured glucose peak time and rate of decline and found this to be in line with the control (non-surgical) group [19]. Another randomised placebo-controlled crossover trial of 18 subjects showed the same antagonist raised the glucose nadir by 21–26% depending on the dose (P.001 and 0.002) and decreased insulin peak by 21–23% (P 0.042 and 0.029) [20]. A third case control study of 24 participants using an antagnoist found that these effects of glucose stabilisation were stabilised more in those that had had MBS than those with no surgery [27].

In terms of GLP-1 receptor agonists (plus the mixed agonist-GIP modulator single case study), this similarly showed reduction in postprandial glucose peak and more appropriate insulin peak and timing after induction the intervention which subsequently responded to improved symptoms (total 7 patients) [29, 30]. Similarly, time spent with blood glucose level < 70 mg/dL was decreased from 12% to 1% in a case report that utilised continuous glucose monitoring after uptitration to full dose of an agonist [22].

#### Qualitative Measures

Other outcomes were quality of life/mood improvement, included as an outcome in 3 case reports and appeared to have positive benefits associated with GLP-1 RMs [22–24]. These were all GLP-1 receptor aognists.

### Adverse Effects

A case series of three patients using GLP-1 receptor agonists reported that one out of three participants developed post prandial vomiting, however this resolved with education on only eating to satiety and subsequently resolved without any dose reduction [15]. One case study of a GLP-1 RM used in conjunction with Acarbose (an alpha-glucosidase inhibitor) reported unintentional weight loss (amount not disclosed) however this stabilised with regular meals [23]. 12 studies (85 participants) did not report on adverse effects [16–22, 24–28].

Further reports on GLP-1 receptor agonists include a case series of five patients reported some potential rebound symptoms in those who either ceased or reduced their GLP1-RA, both of which resolved after recommencing or downtitrating to previous dosing [26]. One experienced subjective palpitations and nausea which transitioned to abdominal pain and nausea on switching from Liraglutide to Exenatide so treatment with GLP1-RM was ceased despite its good results with dumping syndrome (symptoms and frequent hypoglycaemic episodes have subsequently returned). The fifth case in this particular series also experiences nausea and headache when dose of the GLP-11 RA (monotherapy) was uptitrated to aim to cease all hypoglycaemic episodes (having already had markedly reduced). Therapy was switched to a different GLP1-RM and side effects resolved without any further hypoglycaemic episodes [26].

One study of a GLP-1 receptor antagonist found that there were no increased adverse events in the 28 days period the intervention was applied [20]. In another, investigators had to intervene in the placebo group (not the antagonist) due to low plasma glucose levels so although this is not a safety effect profile of the drug itself, it does indicate some risk that should be considered given the recommendations into further research this scoping review provides [19]. The third antagonist study did not comment on side effects or safety [27].

The combined agonist-GIP therapy cited insurance issues with getting Tirzepatide for longer than 6 weeks, after which symptoms of dumping syndrome returned [30].

### Type of Dumping Syndrome

Of the studies meeting inclusion criteria, nine (57 participants or 53.27%) looked at late dumping syndrome, five (49participants or 45.79%) did not report on the kind of dumping syndrome studied and one case study looked at both early and late dumping syndrome [15–22, 24–29].

### Methods of Collecting Data On Dumping Syndrome

Studies utilised variable methods to collect data on diagnosis of dumping syndrome. Nine studies used only a single method, six studies included multiple measurements, and two case reports did not disclose how they collected their data. Unfortunately, the largest study with 27 participants plus one other case report did not disclose how they diagnosed dumping syndrome [15, 28]. However, the next two largest studies (totalling 42 participants) used the Mixed Meal Tolerance Test (MMTT) to diagnose dumping syndrome which was an equal amount of people overall who were diagnosed using symptoms across other studies (also 42) [15–20, 25, 27, 28]). The Oral Glucose Tolerance Test (OGTT) was used in 35 patients through four studies [18, 19, 21, 29]. Then, continuous glucose monitoring was the next most used method of measuring outcomes with 30 total patients [16, 17, 20, 22–26, 30]. Of these, two case reports used flash continuous glucose monitoring where participants were required to manually scan the monitor to get blood glucose levels, and the rest were continuously updating [22, 24]. Finally, point of care testing was completed in six patients although in both cases they had multiple other measures, and self glucose monitoring was measured in the study of 18 people in conjunction with other testing methods [16, 20, 26].

### Type of GLP-1 Modulator Used and Dosing Regimen

Regarding GLP-1 receptor agonists, Liraglutide was the most used in the studies with 45 (42.06%) subjects in total [15, 21, 23–26, 28, 29]. Its dosing regimen ranged from 0.6 mg subcutaneously daily for two days, to up to 3 mg daily. Smaller amounts of cases used Semaglutide (2 case studies), both starting at 0.25 mg weekly and one uptitrating to 0.5 mg weekly after a month of 0.25 mg [16, 22]. 

 For GLP-1 receptor antagonists, a randomised, placebo-controlled crossover study of 18 people (16.82%) used Avexitide after placebo for two weeks, and then were half randomised to at 30 mg twice daily or 60 mg daily first [20]. In another, Ex-9 (7500pmol/kg) infusions which were used in two studies totalling 43 patients (40.19%) [17, 25]. In another, it was used at a dose of 5microg subcutaneously daily and uptitrated to 10 micrograms daily [17].

The dual action GLP-1RA and GIP modulator, Tirzepatide, was used in a single case study at 2.5 mg daily, which was increased to 5 mg daily after an undisclosed period of time for weight loss [30].

### Concurrent Treatment

Four case studies and two case series included concurrent treatment with the GLP1-RM for treatment of dumping syndrome, totalling 36 patients or 33.64% [16, 17, 23, 25, 26, 28]. One case series contained a study where one patient used acarbose and nifedipine, and the other used nifedipine, diazoxide and acarbose [26]. A second case series of 27 patients contained some that also used a low glycaemic diet [28].

## Discussion

This scoping review demonstrates emerging evidence to suggest that GLP-1 RMs may provide some benefit in resolving or reducing not only postprandial hypoglycaemic episodes, a marker of late dumping syndrome, in patients who underwent MBS, but also assist in achieving stabilisation of glucose levels (when compared to its use in non-operative groups) with potential improvement or resolution of symptoms. This contrasts with a recent systematic review by Llewellyn et al., which focused primarily on postprandial hypoglycaemia as an outcome [13]. Furthermore, when the patient experience was considered, there was evidence to suggest that GLP1-RM treatment may lead to an improved quality of life for the MBS patient with dumping syndrome. Specifically, our included studies generally reported transient gastrointestinal upset and nausea which was dose-dependent, suggesting a need for dose-titration and patient education prior to commencement of therapy. Similarly, rebound symptoms upon cessation or down titration suggest a need for maintenance therapy or careful weaning. These results however occur on the background of highly heterogeneous studies, many of which were from case reports and small non-randomised observational studies that lacked power and with scarcely collected outcome data. Therefore, given this paucity in evidence and these challenges, there is at present insufficient evidence to robustly report and draw conclusions on safety and efficacy. It should however be noted that concurrent medications, dietary counselling, dose titration, and changes in meal pattern can themselves improve symptoms and hypoglycaemia which are useful avenues to consider when it comes to future research and the role of confounders. Despite this, our scoping review provides some emerging insights into the use of GLP-1 RMs, including their dosing regimens, associated potential safety and efficacy outcomes as well as issues with access that may assist future studies in assessing the further role of these medications in the guideline-based management of the MBS patient with dumping syndrome.

On initial literature screening, our a priori intent was to examine the role of GLP-1 receptor agonism alone, given the aforementioned potential overlaps between the pathophysiological mechanisms of dumping syndrome and the potential role of GLP-1 receptor aognists in delaying gastric emptying [31]. Despite this, our search yielded studies that also interestingly examined the role of GLP-1 dual modulators (receptor agonism with GIP) and GLP-1 receptor antagonists. Given the iterative nature of scoping review methodology, these articles were decided to be included to allow examination of the potential roles of GLP-1 modulating medications on dumping syndrome in general. On examining these two other classes of medications, in general antagonists were demonstrated to also provide an element of symptomatic relief and glucose stabilisation. Dual modulators were also found to improve symptoms, which recurred upon cessation due to insurance issues. Taken together, it appears that there are multiple mechanisms that may influence the pathogenesis of dumping syndrome (both early and late) in MBS patients. For example, it is suggested that GLP-1 receptor antagonists have an effect on the release of incretins that is associated with the vasomotor and GI symptoms associated with early dumping as well as preventing hyperinsulinaemia that may be associated with late dumping [32]. This is in addition to the known effect of GLP-1 receptor agonists in delaying gastric emptying and therefore preventing the rapid delivery of nutrients into the jejunum to drive dumping syndrome [32]. However, due to the limitations of the underlying evidence, it is perhaps too early to affirm that our findings are a result of these medications alone. Future studies should aim at exploring the various modulatory GLP-1 receptor pathways to further elucidate the effect of antagonism and agonism on dumping syndrome.

When considering the current paradigms in dumping syndrome management, conservative measures such as dietary modification has been associated with some low-level evidence for significant symptomatic improvement [33]. This includes reducing carbohydrates that are rapidly absorbed and ensuring diet consists of high fibre and protein and is consumed slowly [11]. In the case where there is failure of dietary measures in managing dumping syndrome, other therapies such as acarbose (an a-glycosidase hydrolase inhibitor, slowing intestinal transit) are known to improve late dumping syndrome hypoglycaemia in 18–50% of patients [11]. However, acarbose use has higher reports of side effects up to 76% (such as flatulence, abdominal pain, bloating, nausea and diarrhoea) when compared to those of GLP-1 RMs, which most common reported side effects include nausea (up to 50% of people), vomiting, diarrhoea, constipation and abdominal pain [33, 34]. Despite initial concerns, there has been no direct cause and effect between GLP-1 RMs and pancreatitis, pancreatic or medullary cancer with methodological flaws in initial research positing this, however it should be avoided in patients who are considered at high risk of these diseases [35]. Somatostatin analogues are proposed as another option through multimodal pharmacological mechanisms along the dumping syndrome pathway, ultimately improving both early and late dumping syndrome. However, for late dumping syndrome, somatostatin analogue injections are required up to three times daily (compared with Liraglutide, the most common GLP-1 RM in this scoping review, which is once per day), and the out-of-pocket costs are significant in many jurisdictions (in Australia, therapies can amount to approximately AUD$120/day somatostatin analogues AUD$7.60 Liraglutide) [36]. Aside from the gastrointestinal side-effects like nausea and diarrhoea, somatostatin analogues can also predispose patients to gallstone formation which is particularly relevant in those who have had Roux-en-Y for risk of choledocholithiasis [37]. Finally, surgical reversal or continuous enteral feeding has been done in those with failure to respond to all above treatment options, however these were low level evidence (grade C level IV) and some showed poor outcomes on reducing dumping syndrome regardless of approach and some associated surgical complications [37].

Finally, the consideration of GLP1-RMs as a therapeutic intervention for dumping syndrome contributes to the increasing directions for further research. Aside from management of obesity and cardiovascular risk reduction, many novel uses are being explored in literature including use in regulation of androgen production in polycystic ovarian syndrome, liver disease, neurodegenerative disease, substance use disorders, obstructive sleep apnoea and sleep disordered breathing issues among others [38–40]. Currently issues outlined by literature include side effects, perioperative management requiring prolonged fasting, regain of weight after cessation and financial and ethical equity. Future research into its use (including but not limited to post MBS dumping syndrome) should be complemented by investigation into how to overcome these barriers to secure its therapeutic future [38].

## Limitations

This scoping review included a modest sample size and lacked randomised control trials. Therefore, the studies which were analysed were heterogeneous and poorly powered, with a paucity in the evidence that did not allow for clinically significant subgroup analyses, such as by BMI, amount of weight lost, patient comorbidities and by late or early dumping syndrome to occur. International guidelines posit that a spontaneous glucose plasma level of < 2.8mmol/l indicates late dumping syndrome, and that early dumping syndrome is positive for a modified OGTT if after 30 min there is an increase in haematocrit level by > 3% or an increased heart rate > 10 beats per minute (BPM) [11]. The authors acknowledge that there was heterogeneity in diagnosis of dumping syndrome (both in investigation and definition of including early/late) across included papers in this review. Those that did use the OGTT for diagnosis of dumping syndrome did not always detail the structure of these tests, and international consensus is that this test does not have good reproducibility anyway [11]. Essentially, investigations for diagnosis are variable which was reflected across the literature gathered. Hence caution should be used when comparing these studies and it should not be assumed that the outcomes measured were uniformly beneficial.

Furthermore, many included studies used either preceding or concurrent treatment to manage dumping syndrome so these outcomes cannot be completely attributed to the GLP-1 RM alone. Given the novel nature of this class of drug, there was also heterogeneity in the GLP-1 RM regimen, dosage and administration time between studies. Furthermore, there was a paucity of evidence regarding side effects in the studies included. Other current barriers to gaining treatment with GLP-1 RMs include both legislative and financial limitations in Australia (despite being significantly cheaper than somatostatin analogues currently utilised as management of dumping syndrome).

It should also be noted that the weight loss effect of GLP-1 RM can be a drawback in patients who have undergone MBS, especially those who have lost weight successfully post operatively and may be low BMI.

## Conclusion

The purpose of this scoping review was to review existing literature on GLP-1 RMs in the management of dumping syndrome, a significant cause of morbidity and impaired quality of life for patients who experience this complication after MBS. From the included studies, we found preliminary evidence suggesting a potential role for GLP-1 RMs in the management of both early and late dumping syndrome. Current management of dumping syndrome is limited in both evidence and effect, with more reported side effects and treatment having more side effects, needing more regular dosing and being more expensive. Rather than viewing the pooling of heterogenous data and differing outcomes as a methodological weakness, this scoping review demonstrates there is an opportunity for high-quality, meaningful research following international guidelines and utilising GLP-1 RMs in yet another novel therapeutic way. Duration of benefit, safety and adherence should also be assessed in future studies of GLP-1 RMs for use in dumping syndrome post MBS, as well as broader research such as equity, cessation of treatment effects and perioperative management.

## Data Availability

No new data was generated from this manuscript. Data was obtained and cited appropriately from included studies in references.

## Appendix A: Complete search strategy

### Embase Classic+Embase <1947 to 2025 May 05>

1. exp glucagon like peptide 1 receptor agonist/60446
2. ((glucagon like peptide 1 receptor* or glucagon like peptide one receptor* or GLP-1RA*) adj3 (agonist* or antagonist* or medication* or drug* or agent* or analog*)).mp.19078
3. (semaglutide or liraglutide or exenatide or tirzepatide or albiglutide or dulaglutide or lixisenatide or Ozempic or Wegovy or Rybelsus or Saxenda or Byetta or Adlyxin or Trulicity or Mounjaro).mp. 26731
4. 1 or 2 or 3 61481
5. exp*dumping syndrome/346
6. (((‘dumping syndrome’ or ‘dumping) adj3 syndrome’) or ‘postprandial hypoglyc*’ or ‘late dumping’ or ‘early dumping’).mp. [mp=title, abstract, heading word, drug trade name, original title, device manufacturer, drug manufacturer, device trade name, keyword heading word, floating subheading word, candidate term word] 4179
7. 5 or 6 4179
8. 4 and 7217

### Ovid MEDLINE(R) Epub Ahead of Print and In-Process, In-Data-Review & Other Non-Indexed Citations and Daily

1. exp glucagon like peptide 1 receptor agonist/ 8479
2. ((glucagon like peptide 1 receptor* or glucagon like peptide one receptor* or GLP-1RA*) adj3 (agonist* or antagonist* or medication* or drug* or agent* or analog*)).mp. 7730
3. (semaglutide or liraglutide or exenatide or tirzepatide or albiglutide or dulaglutide or lixisenatide or Ozempic or Wegovy or Rybelsus or Saxenda or Byetta or Adlyxin or Trulicity or Mounjaro).mp.11670
4. 1 or 2 or 3 15825
5. exp *dumping syndrome/1276
6. (((‘dumping syndrome’ or ‘dumping) adj3 syndrome’) or ‘postprandial hypoglyc*’ or ‘late dumping’ or ‘early dumping’).mp. [mp=title, book title, abstract, original title, name of substance word, subject heading word, floating sub-heading word, keyword heading word, organism supplementary concept word, protocol supplementary concept word, rare disease supplementary concept word, unique identifier, synonyms, population supplementary concept word, anatomy supplementary concept word] 3018
7. 5 or 6 3018
8. 4 and 717

**EBM Reviews - Cochrane Database of Systematic Reviews <2005 to April 28, 2025>**

**EBM Reviews - ACP Journal Club <1991 to April 2025>**

**EBM Reviews - Database of Abstracts of Reviews of Effects <1st Quarter 2016>**

**EBM Reviews - Cochrane Clinical Answers <April 2025>**

**EBM Reviews - Cochrane Central Register of Controlled Trials <March 2025>**

**EBM Reviews - Cochrane Methodology Register <3rd Quarter 2012>**

**EBM Reviews - Health Technology Assessment <4th Quarter 2016>**

**EBM Reviews - NHS Economic Evaluation Database <1st Quarter 2016>**

1. exp glucagon like peptide 1 receptor agonist/ 0
2. ((glucagon like peptide 1 receptor* or glucagon like peptide one receptor* or GLP-1RA*) adj3 (agonist* or antagonist* or medication* or drug* or agent* or analog*)).mp. 1378
3. (semaglutide or liraglutide or exenatide or tirzepatide or albiglutide or dulaglutide or lixisenatide or Ozempic or Wegovy or Rybelsus or Saxenda or Byetta or Adlyxin or Trulicity or Mounjaro).mp. 6284
4. 1 or 2 or 3 6567
5. exp*dumping syndrome/7
6. (((‘dumping syndrome’ or ‘dumping) adj3 syndrome’) or ‘postprandial hypoglyc*’ or ‘late dumping’ or ‘early dumping’).mp. [mp=ti, ab, tx, kw, ct, ot, fx, sh, hw] 283
7. 5 or 6 283
8. 4 and 78

### Ovid Emcare <1995 to 2025 Week 18>

1. exp glucagon like peptide 1 receptor agonist/16125
2. ((glucagon like peptide 1 receptor* or glucagon like peptide one receptor* or GLP-1RA*) adj3 (agonist* or antagonist* or medication* or drug* or agent* or analog*)).mp. 6205
3. (semaglutide or liraglutide or exenatide or tirzepatide or albiglutide or dulaglutide or lixisenatide or Ozempic or Wegovy or Rybelsus or Saxenda or Byetta or Adlyxin or Trulicity or Mounjaro).mp.7504
4. 1 or 2 or 3 16385
5. exp*dumping syndrome/50
6. (((‘dumping syndrome’ or ‘dumping) adj3 syndrome’) or ‘postprandial hypoglyc*’ or ‘late dumping’ or ‘early dumping’).mp. [mp=title, abstract, heading word, drug trade name, original title, device manufacturer, drug manufacturer, floating subheading word, device trade name, keyword heading word, candidate term word] 529
7. 5 or 6 529
8. 4 and 745

### University of Melbourne Full Text Journals

1. exp glucagon like peptide 1 receptor agonist/0
2. ((glucagon like peptide 1 receptor* or glucagon like peptide one receptor* or GLP-1RA*) adj3 (agonist* or antagonist* or medication* or drug* or agent* or analog*)).mp. 1275
3. (semaglutide or liraglutide or exenatide or tirzepatide or albiglutide or dulaglutide or lixisenatide or Ozempic or Wegovy or Rybelsus or Saxenda or Byetta or Adlyxin or Trulicity or Mounjaro).mp.2402
4. 1 or 2 or 3 2986
5. exp*dumping syndrome/0
6. (((‘dumping syndrome’ or ‘dumping) adj3 syndrome’) or ‘postprandial hypoglyc*’ or ‘late dumping’ or ‘early dumping’).mp. [mp=title, abstract, full text, caption text] 786
7. 5 or 6 786
8. 4 and 7 38

### Google Scholar

GLP-1 agonists and dumping syndrome
